# Supplementary material for: Proteomic Analysis Reveals That Mitochondria Dominate the Hippocampal Hypoxic Response in Mice
Source: Int J Mol Sci. 2022 Nov 15;23(22):14094. doi: 10.3390/ijms232214094 (PMC9697535; doi:10.3390/ijms232214094)
Supplement: Supplementary file 1 [file ijms-23-14094-s001.zip › Supplementary figures-ijms-1934583.pdf]

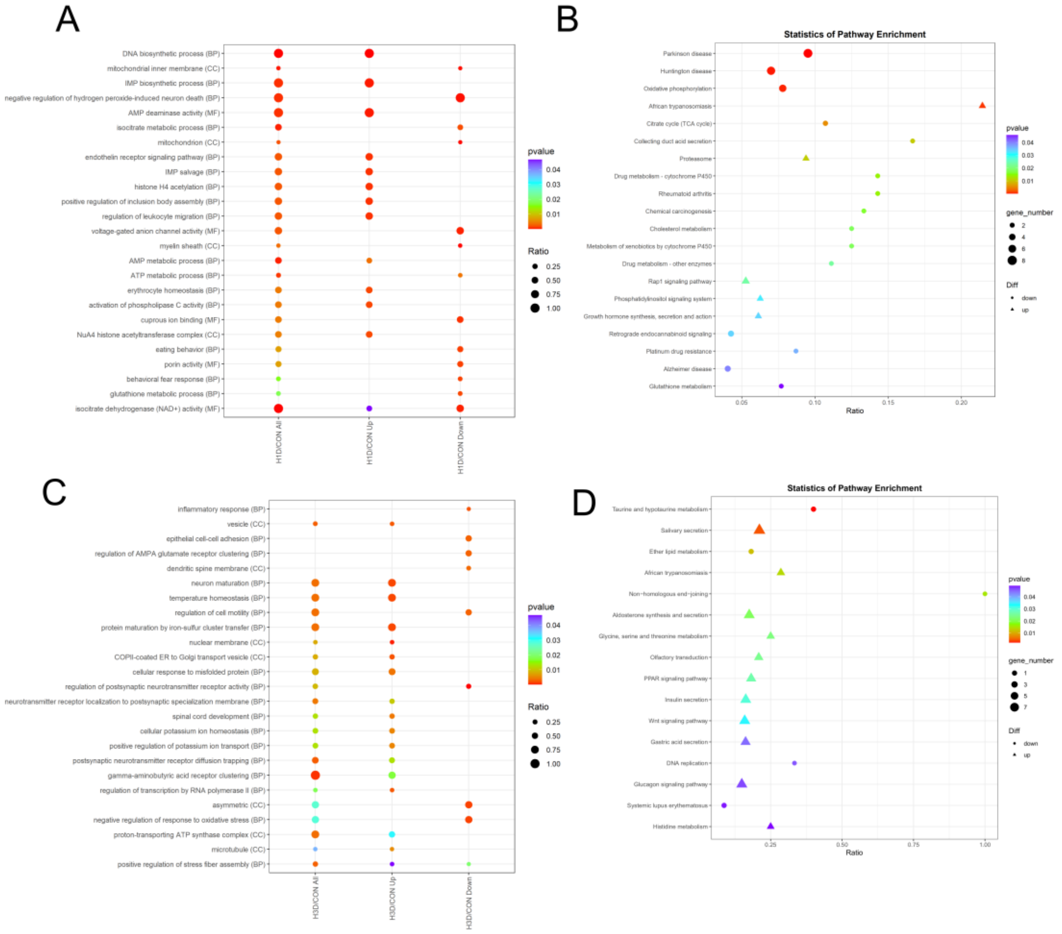

Supplementary Figure S1. (A, B) GO graphs classified by cellular component, molecular function, and biological process (A) and KEGG pathway (B) in H1D mice compared with CON mice. (C, D) GO graphs classified by cellular component, molecular function, and biological process (C) and KEGG pathway (D) in H3D mice compared with CON mice.

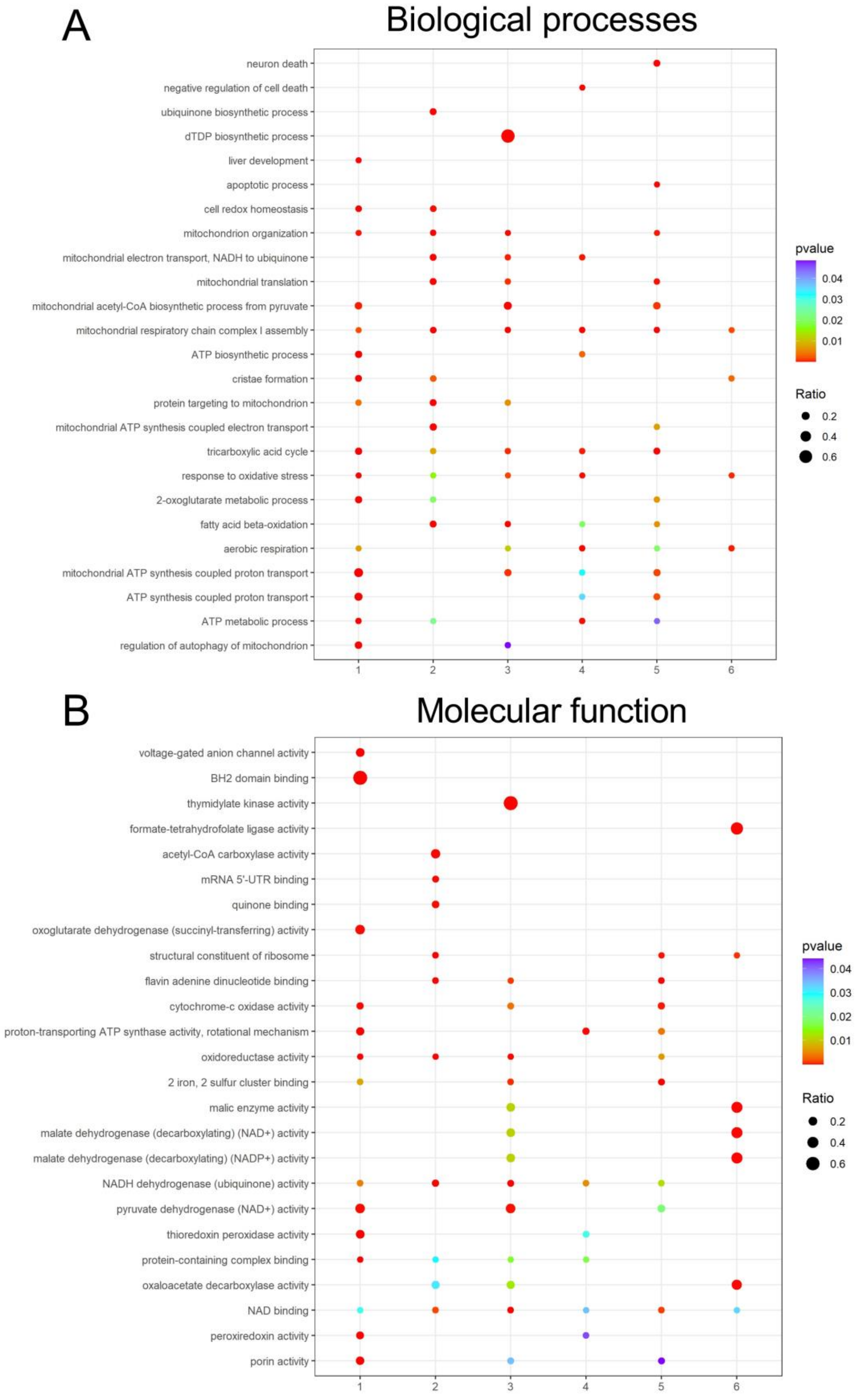

Supplementary Figure S2. (A, B) Top 20 GO items screened by biological process (A) and molecular function (B) in mitochondria.
